# Inferring phenotypes from substance use via collaborative matrix completion

**DOI:** 10.1186/s12918-018-0623-5

**Published:** 2018-11-22

**Authors:** Jin Lu, Jiangwen Sun, Xinyu Wang, Henry Kranzler, Joel Gelernter, Jinbo Bi

**Affiliations:** 1 Department of Computer Science and Engineering, University of Connecticut, 371 Fairfield Way, Unit 4155, Storrs, CT USA; 2 Department of Psychiatry, University of Pennsylvania Perelman School of Medicine, 3535 Market Street, Suite 500 and Crescenz Veterans Affairs Medical Center, Philadelphia, PA USA; 3 Departments of Psychiatry, Genetics, and Neurobiology, Yale University School of Medicine, 333 Cedar St, New Haven, CT USA

**Keywords:** Phenotype imputation, Matrix completion, Addiction, Substance use disorder, Parallel computing

## Abstract

**Background:**

Although substance use disorders (SUDs) are heritable, few genetic risk factors for them have been identified, in part due to the small sample sizes of study populations. To address this limitation, researchers have aggregated subjects from multiple existing genetic studies, but these subjects can have missing phenotypic information, including diagnostic criteria for certain substances that were not originally a focus of study. Recent advances in addiction neurobiology have shown that comorbid SUDs (e.g., the abuse of multiple substances) have similar genetic determinants, which makes it possible to infer missing SUD diagnostic criteria using criteria from another SUD and patient genotypes through statistical modeling.

**Results:**

We propose a new approach based on matrix completion techniques to integrate features of comorbid health conditions and individual’s genotypes to infer unreported diagnostic criteria for a disorder. This approach optimizes a bi-linear model that uses the interactions between known disease correlations and candidate genes to impute missing criteria. An efficient stochastic and parallel algorithm was developed to optimize the model with a speed 20 times greater than the classic sequential algorithm. It was tested on 3441 subjects who had both cocaine and opioid use disorders and successfully inferred missing diagnostic criteria with consistently better accuracy than other recent statistical methods.

**Conclusions:**

The proposed matrix completion imputation method is a promising tool to impute unreported or unobserved symptoms or criteria for disease diagnosis. Integrating data at multiple scales or from heterogeneous sources may help improve the accuracy of phenotype imputation.

## Introduction

Substance use disorders (SUDs) are common, complex diseases that are difficult to treat and impose a substantial public health burden. According to the 2015 National Survey on Drug Use and Health [1], there were 27.1 million Americans (10.1% of total) aged 12 or older who used an illicit drug in the past 30 days and approximately 7.7 million who had a SUD related to the use of illicit drugs. Alcohol use is even more common with approximately 138.3 million Americans aged 12 or older reporting current use and 15.7 million suffering from an alcohol use disorder (AUD). Substance use can lead to a wide range of health problems, including toxic effects (e.g., fatal overdose), other effects of intoxication (e.g., accidental injury) and diseases due to chronic exposure, such as cirrhosis of the liver, blood-borne infection (e.g., HIV) and mental disorders (e.g., psychosis) [2]. It was estimated that about 300,000 deaths attributable to SUDs in 2015 worldwide [3], and 0.7*%* of global disability-adjusted life years (DALYs) attributable to SUDs in 2015 [4]. The effectiveness of treatments for SUDs is limited, in part due to an inadequate understanding of their genetic basis, which limits medications development. To date, there has been limited success in the identification of variation contributing to risk of SUDs through genome-wide association studies (GWASs) [5].

As complex polygenic disorders, SUD risk is attributable to many genetic variants of small effect size. GWASs have been limited by the small size of study populations available for analysis [6], which determine the statistical power of an association test in GWAS [7]. One approach to increasing the sample size is to aggregate samples from multiple GWASs [8, 9]. However, subjects aggregated from different studies often have missing phenotypic information, such as diagnostic criteria for a specific SUD because it may not have been a target of the original study. The lack of phenotyic assessment is usually handled by removing these subjects from the aggregated association analysis [8, 9], further reducing statistical power.

In this paper, we explore the use of a machine learning approach to infer missing phenotypes for a subject. The premise of this statistical inference method is that many different SUDs share common neurobiological processes, including those that mediate reward, behavioral control, and anxiety or stress responses [10]. In addition, people with SUDs often use multiple substances so different SUDs often co-occur. For example, heroin addicts applying for methadone treatment in the United States are regular users of alcohol (50%), benzodiazepines (33%), cocaine (47%), and marijuana (69%) [11].

**Fig. 1:**
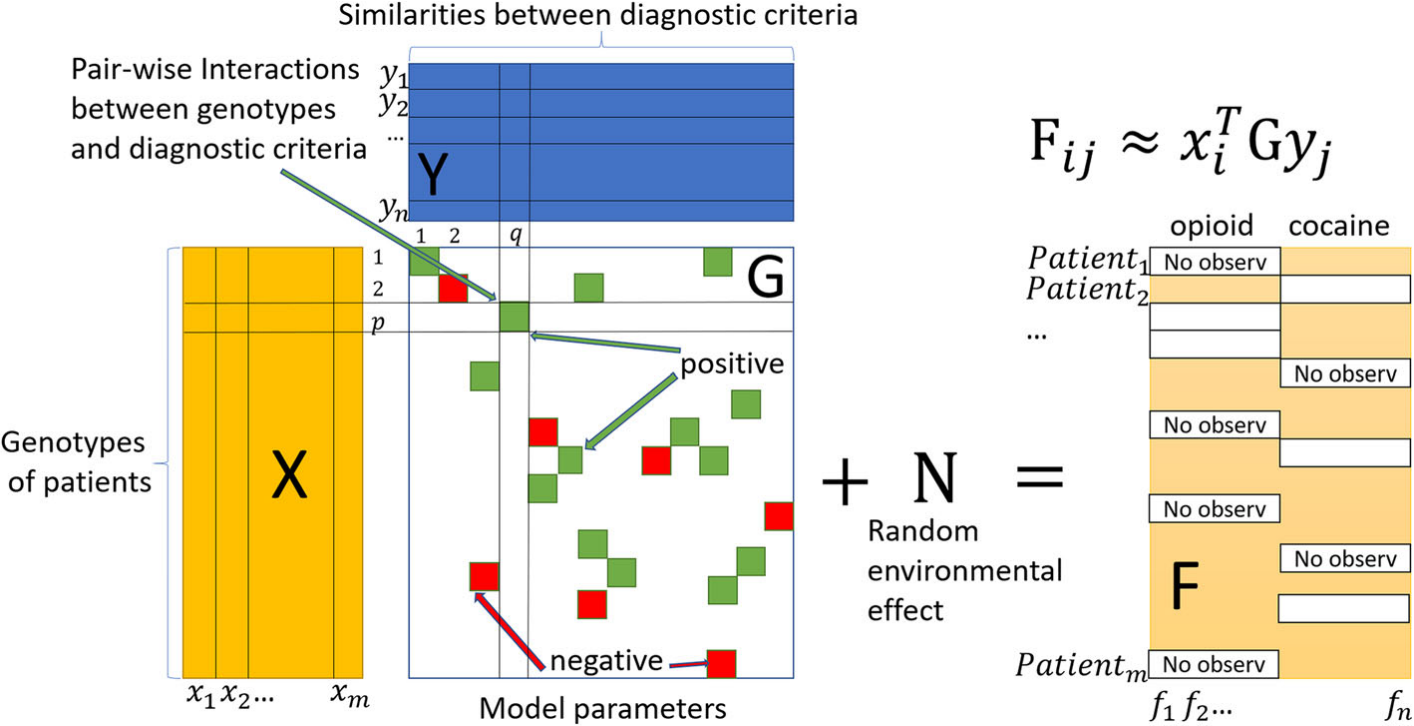
Inferring phenotypes for diagnoses of substance use disorders (e.g., opioid and cocaine illustrated here) via matrix completion. Phenotypes for a set of patients are organized into a matrix **F** with rows for *m* patients and columns for *n* diagnostic symptoms. Related features that describe patients and symptoms are available, such as, genotypes of individuals in the matrix **X** and pair-wise similarities between diagnostic symptoms in the matrix **Y**. A bi-linear model: **F**=**X**^*T*^**GY**+**N** is used where **G** and **N** are model parameters to be learned, and quantify the impact of genotype-symptom interactions and the residual from any other effect (mainly random environmental effect), respectively

Phenotype inference is analogous to a recommender system that predicts the preference (endorsement) of a user (patient) to a product (symptom) based on known preferences of other related products (related symptoms). A recommender system is based on an assumption that similar users give similar ratings to similar products. Analogously, similar patients (e.g. those sharing a certain portion of their genetic background) may endorse similar symptoms for biologically correlated disorders. The correlations among symptom endorsements are the basis for drawing the inferences regarding missing phenotypes. Matrix completion methods are widely used to infer missing ratings in a recommender system by organizing the ratings of different users (rows) for various products (columns) into a matrix. By organizing the phenotypes of patients related to disorder(s) into a matrix (as shown in Fig. 1), we can impute the missing phenotypes by completing the matrix.

Classic matrix completion methods [12, 13] assume that the matrix to be completed is low rank because of the rating correlations, and fill in the missing entries with values that lead to a completed matrix that yields a minimal rank. These methods do not consider what we call the side information, such as genetic composition of patients and characteristic of disorders, which can be very informative to the data completion. Even though recently side information has been considered in a few advanced matrix completion methods [14, 15], many of these methods have non-convex formulations, resulting in very difficult optimization problems [16–19].

To address these issues, we have recently developed a method that completes a matrix by building a bi-linear predictive model with two side feature matrices, one describing the row entities of the matrix (e.g. patients) and the other describing the column entities (e.g. disease symptoms) [20]. The optimization problem in this method is convex, and thus is easier to solve comparing to non-convex formulations. This method has a provable recovery guarantee that the true matrix can be recovered as long as there are *O*(*log N*) observed entries (where *N* is either the number of rows or columns whichever is greater), when the side information spans the full latent space of the matrix. When otherwise, a formula is derived to give the number of observed entries that is necessary to achieve *ε*-recovery (e.g. recovery error no bigger than *ε*). A limitation of this work is that the algorithm developed for solving the optimization problem lacks scalability to large datasets, whereas large numbers of dimensions are very common in genetic studies. Thus, in this paper, we propose a new parallel and stochastic algorithm to solve the optimization problem proposed in [20]. This algorithm converges to its global optimum with a sub-linear rate.

Figure 1 illustrates how we infer the missing phenotypes. The matrix **F** contains the phenotypes to be completed where rows represent different patients and columns correspond to phenotypes (e.g., diagnostic criteria for SUDs), respectively; **X** is a matrix consisting of genetic data of patients; **Y** is a matrix composed by pair-wise similarities between diagnostic criteria. In our model, **F** is assumed to be given by **X**^*T*^**GY**+**N** and the missing entries are inferred by learning the two model parameter matrices **G** and **N**. Here, **N** is used to fit the random environmental effect on phenotypes. In our evaluation, we used an **X** that contains data for the genetic variants pre-identified by a GWAS. Our approach was first validated in a set of simulations, and then used to analyze an aggregated SUD dataset. We also compared our approach against several other recent matrix completion methods.

The following notation is used throughout the paper. A bold lower case letter denotes a vector as **v** and ∥**v**∥_*p*_ reflects the *ℓ*_*p*_-norm of the vector **v** by ∥**v**∥_*p*_=(|**v**_(1)_|^*p*^ + ⋯ + |**v**_(*d*)_ |^*p*^) ^1/*p*^, where **v**_(*i*)_ is the *i*-th entry in **v** and *d* represents the number of elements in **v**. A bold upper case letter represents a matrix as **M**_*n*×*d*_ with the size of *n*-by-*d*. ∥**M**∥_*F*_ computes the Frobenius norm of **M** and *tr*(**M**) computes its trace.

## Methods

### Materials

∙*Subjects.* A total of 7189 subjects were aggregated from three family-based or case-control genetic studies of cocaine use disorder (CUD) and opioid use disorder (OUD). Subjects were recruited at five sites: Yale University School of Medicine (N =3348, 46.57%), the University of Connecticut Health Center (N = 2407, 33.48%), the University of Pennsylvania Perelman School of Medicine (N =955, 13.28%), the Medical University of South Carolina (N =276, 3.84%), and McLean Hospital (N = 203, 2.82%). The institutional review board at each site approved the study protocol and informed consent forms. The National Institute on Drug Abuse and the National Institute on Alcohol Abuse and Alcoholism each provided a Certificate of Confidentiality to protect participants. Subjects were paid for their participation. Of the 7189 subjects, 7008 self-reported having used cocaine and were included in a GWAS of CUD [9]; 4843 self-reported having used an opioid and were included in a GWAS of OUD [21]. In total, 4662 subjects self-reported having used both cocaine and opioids; of that number, 3441 subjects who in their lives had used opioids and cocaine more than 11 times were included in the evaluation of the proposed approach to infer cocaine and opioid use behaviors. Statistics describing these datasets can be found in Table 1.

**Table 1:** Sample size by study and race: African-Americans (AAs) and European-Americans (EAs)

	AAs	EAs
CUD association, microarray	2718	2037
CUD association, exome sequencing	940	1395
OUD association, microarray	1398	1756
OUD association, exome sequencing	540	1190
Phenome inference	1149	2292

Our sample included 1645 subjects from 740 small nuclear families (SNFs) and 5544 unrelated individuals. The self-reported population distribution of the sample was 45.51% European-American (EA), 50.65% African-American (AA), and 3.83% other race. The majority of participants (59.76%) were never married; 28.22% were widowed, separated, or divorced; and 12.02% were married. Few subjects (0.07%) had only a grade school education; 40.41% had some high school, but no diploma; 27.90% completed high school only; and 31.45% received education beyond high school.

∙*Assessments.* Phenotypic information was assessed through administration of the Semi-Structured Assessment for Drug Dependence and Alcoholism (SSADDA), a computer-assisted interview comprised of 26 sections (including sections for both cocaine and opioid use) that yielded diagnoses of various SUDs and Axis I psychiatric disorders, as well as antisocial personality disorder [22, 23]. The diagnostic reliability for both DSM-4 [24] cocaine dependence (CD) and opioid dependence (OD) were excellent, with test-retest reliability *κ*=0.92 for CD and 0.94 for OD, and inter-rater reliability *κ*=0.83 for CD and 0.91 for OD [22]. The reliability of the individual criteria ranged from *κ*=0.47−0.60 for CD and *κ*=0.56−0.90 for OD. To assist in the diagnosis of CUD, OUD or SUD, the DSM-5 lists 11 criteria, which can be clustered into four groups: impaired control, social impairment, risk use and pharmacological criteria. The criteria related to CUD and OUD were evaluated using questions from the SSADDA cocaine and opioid sections, respectively. In this study, we impute the missing data for the 11 criteria of CUD and OUD, respectively, for subjects who had no prior exposure to either cocaine or opioid. Specifically, we impute CUD criteria from OUD criteria, or vice-versa, using genotypic data. To evaluate the proposed method against the observed ground truth, in our experiments we used the 3441 subjects for whom we had both CUD and OUD criteria.

Subjects were genotyped using one of the following two methods: the Illumina HumanOmni1-Quad v1.0 microarray (MA) (*N*=4281) and Infinium CoreExome-24 Kit microarray (EMA) (*N*=2450) see Table 1 for data statistics. Detailed descriptions of the genotyping and variant calling procedures are available [8, 9]. Genotypes were imputed with IMPUTE2 [25] using the genotyped variants and the 1000 Genomes reference panel (www.internationalgenome.org; released June 2011) (1000 Genomes Project Consortium, 2010). For both genotyping samples, a total of 47,104,916 variants were imputed. We used only the variants with an imputation quality score ≥0.99.

### Analysis

Our analysis was conducted in two steps. We first identified candidate genetic variants that were nominally associated with either CUD or OUD by a GWAS, which were subsequently used as side features in matrix completion to infer missing phenotypes.

#### Candidate genetic variants for CUD and OUD

The genetic relationship (GR) between each pair of subjects was evaluated with LDAK4 [26]. The evaluation was done separately for the MA and EMA samples, and included only common variants with minor allele frequency (MAF) ≥0.03 and a very high IMPUTE2 quality score ≥0.99. There were 3,140,006 single nucleotide polymorphisms (SNPs) for MA and 604,884 SNPs for EMA included in the GR estimation. The estimated GR matrix containing the GR values of each pair of subjects was used in the subsequent association analysis to account for the population effect from genetic correlations.

To verify and correct the misclassification of self-reported race, we compared the MA (and EMA) data of all subjects with the genotypes from the HapMap 3 reference population: CEU, YRI, and CHB. To characterize the genetic architecture of the sample, we conducted a principal component (PC) analysis in the sample using PLINK [27] and 489,697 SNPs (and 91,089 SNPs) that overlapped between the HapMap panel and those included in the GR evaluation in the MA (and EMA) datasets (after pruning the SNPs for linkage disequilibrium (LD), defined as *r*^2^>80%). The first PC scores distinguished AAs and EAs, for which association analysis was done separately. The first three PCs were used in the analysis of each population to correct for residual population stratification.

The CUD (or OUD) criterion count is the number of the 11 diagnostic criteria endorsed by a subject, and was used in the GWAS to identify genetic variants. We used the genome-wide efficient mixed model association (GEMMA) method [28] to perform association tests with sex and age as covariates. We combined the results from all eight studies (with the two different traits [CUD or OUD], datasets [MA or EMA], and populations [AAs or EAs]) in a meta analysis using METAL [29]. Genetic variants with meta *P*-value <1×10^−5^ were used as candidate variants (i.e., side features) in the phenotype inference process.

#### Matrix completion

Matrix completion techniques are commonly used in recommender systems to ‘complete’ the user-product rating matrix with only a fraction of available ratings. Classic matrix completion methods commonly assume that the true underlying matrix is low rank. Low rank matrix completion methods [12, 13] solve the following problem:

1 $$ \min_{\mathbf{E}} \|\mathbf{E}\|_{*},~~~ \text{subject to} ~~~ R_{\Omega}(\mathbf{E})=R_{\Omega}(\mathbf{F}),  $$

where $\mathbf {F} \in \mathbb {R}^{m \times n}$ is the partially observed low rank matrix (with a rank of *r*) that requires recovery, *Ω*⊆{1,⋯,*m*}×{1,⋯,*n*} be the set of indexes of the observed components in **F**, the mapping *R*_*Ω*_(**M**): $\mathbb {R}^{m \times n} \rightarrow \mathbb {R}^{m \times n}$ gives another matrix whose (*i*,*j*)-th entry is **M**_*i*,*j*_ if (*i*,*j*)∈*Ω* but 0 otherwise, and ∥**E**∥_∗_ computes the nuclear norm of **E**.

Several publications [14, 15] propose non-convex matrix factorization formulations to utilize side information. These methods usually have no theoretical guarantees. Alternatively, others propose convex formulations with provable guarantees on matrix recovery [17–19]. All these methods construct a bi-linear model **X**^*T*^**GY** that satisfies *R*_*Ω*_(**X**^*T*^**GY**)=*R*_*Ω*_(**F**) where $\mathbf {X}_{d_{1} \times m}$ contains *d*_1_ features that describe the *m* row entities and $\mathbf {Y}_{d_{2} \times n}$ contains *d*_2_ features that describe the *n* column entities of **F**. In an early work [17], the side feature matrices **X** and **Y** are assumed to be orthonormal and locate, respectively, in the latent column and row spaces of **F** to prove exact recovery (i.e., recovery of the true matrix) with a reduced sampling rate in comparison with the matrix completion without side features. Another method proposed in [18] achieves an *ε*-recovery with provable low sampling rate when the side features are noisy and exact recovery would not be possible. This method extends the above inductive model by adding a term **N** (in other words, using **X**^*T*^**GY**+**N**), and the matrix **N** is assumed to be low rank. In all these methods, **G** is required to be low rank to obtain a low rank **E**:**E**=**X**^*T*^**GY**[+**N**] that approximates the low rank **F**. Mathematically, however, a low rank **E** does not necessarily imply a low rank **G** so the requirement of low rank **G** is unnecessary. Thus, we proposed a method in [20] that eliminates this requirement, and we use this method here to complete the diagnostic criterion matrix. We first briefly review the method in [20], then introduce a novel parallel and stochastic algorithm for solving the optimization problem in this method.

#### Matrix completion with side information

We predict an entry *f* (e.g., the symptom for the *i*-th patient and the *j*-th diagnostic criterion) in **F** based on the side feature (column) vectors **x** for the *i*-th patient and **y** for the *j*-th diagnostic criterion. Note that **x** is a column in **X** and **y** is a column in **Y**. Specifically, our model is *f*=**x**^*T*^**Hy**+**x**^*T*^**u**+**y**^*T*^**v**+*γ*, where **u**, **v**, *γ* and **H** are model parameters. This model uses not only the linear terms **x**^*T*^**u**+**y**^*T*^**v** but also the interaction term **x**^*T*^**Hy**. By defining $\bar {\mathbf {x}} = \left [\mathbf {x}^{T} ~1\right ]^{T}$, $\bar {\mathbf {y}} = \left [\mathbf {y}^{T} ~1\right ]^{T}$ and $\mathbf {G}^{(a=d_{1}+1) \times (b=d_{2}+ 1)} = \left (\begin {array}{ll} \mathbf {H} & \mathbf {u} \\ \mathbf {v}^{T} & \gamma \end {array}\right)$, the above equation can be simplified to: $f = \bar {\mathbf {x}}^{T} \mathbf {G} \bar {\mathbf {y}}$. We solve the following overall optimization problem for the best **G**:

2 $$ \begin{aligned} &\min_{\mathbf{G}, \mathbf{E}} \frac{1}{2}\left\|{\mathbf{X}^{T}\mathbf{G}\mathbf{Y}-\mathbf{E}}\right\|^{2}_{F}+ \lambda_{E} \|\mathbf{E}\|_{\ast} + \lambda_{G} g({\mathbf{G}}), \\ & \text{subject to} R_{\Omega}(\mathbf{E})=R_{\Omega}(\mathbf{F}), \end{aligned}  $$

where **E** is a completed version of **F**. The **X** and **Y** here are two matrices that are created by stacking one row of all ones to the original **X** and **Y**, respectively. To simplify the notation, we use **X** and **Y** to represent the two augmented matrices. Because the phenotype data matrix is expected to be low rank, we also require **E** to be low rank, which is commonly translated into a minimization of the nuclear norm ∥**E**∥_∗_. Additionally, *g*(**G**) is a function of **G** that applies certain priori on **G**. Because side features can be noisy and not all of them and their interactions are helpful in the prediction of **F**, we expect **G** to be sparse and implement *g*(**G**) with ∥**G**∥_1_. The hyperparameters *λ*_*E*_ and *λ*_*G*_ help to balance the three components in the objective function of (2) and can be determined by cross validation.

The formulation (2) differs from the existing methods that make use of side information for matrix completion in several ways. First, existing methods [16–18] solve the problem by finding the optimal bi-linear term $\hat {\mathbf {H}}$ that minimizes ∥**H**∥_∗_ subject to *R*_*Ω*_(**X**^*T*^**HY**)=*R*_*Ω*_(**F**); we expand it to include the linear term within the interactive model. Second, the proposed model adds the flexibility to consider both linear and quadratically interactive terms, and allows the algorithm to determine the terms that should be used in the model by enforcing the sparsity in **G**. Third, existing methods all control the rank of **G** (e.g. by minimizing ∥**G**∥_∗_) to incorporate the prior of low rank **E** (and thus low rank **F**) in their formulations, because **E**=**X**^*T*^**GY** and the rank of **G** bounds that of **E** from above. However, when the rank of **G** is not properly chosen during the tuning of hyperparameters, it may not be a sufficient condition to ensure low rank **E** (if rank(**E**) ≪ the pre-specified rank(**G**)). It is easy to see that besides **G** a low rank **X** or **Y** can lead to a low rank **E** as well. Requiring a low rank condition for **H** or **G** may limit the search space of the interactive model and thus impair prediction performance on the missing entries, which is demonstrated in our empirical results. Moreover, when *λ*_*G*_ is sufficiently large, Eq. (2) is reduced to a matrix completion problem without side information because **G** may be degenerated into a zero matrix. Thus, our formulation is still applicable when there is no access to useful side information.

### Algorithm

In this section, we derive an algorithm to solve Eq. (2) based on the so-called Linearized Alternating Direction Method of Multipliers (LADMM). A stochastic version of the LADMM (StoLADMM) is developed that solves a subproblem at each iteration by randomly selecting a subset of constraints in Eq. (2). Inspired by the stochastic gradient descent algorithm for large scale optimization, stochastic versions of ADMM have recently been investigated [30–33]. However, to the best of our knowledge, ADMM methods with stochastic constraints rather than stochastic objective functions have not been previously discussed, which distinguishes our algorithm from other related works. Besides the major advantage of computational efficiency and the scalability on constraints, when carefully designed, our algorithm has a convergence rate of $O\left (1/\sqrt {k}\right)$ in expectation.

We first show that the LADMM is applicable to our problem and then derive StoLADMM steps.

To use LADMM, the variables to be determined in the optimization problem should be grouped into separate blocks. We use change of variables to meet this condition. We first define **C**=**E**−**X**^*T*^**GY** and plug it into Eq. (2). Following the LADMM scheme, the augmented Lagrangian function of (2) can be written as

$${\begin{aligned} \mathcal{L}(\mathbf{E}, \mathbf{G}, \mathbf{C}, \mathbf{M}_{1}, \mathbf{M}_{2}, \beta) =& \frac{1}{2}\|\mathbf{C}\|^{2}_{F}+\lambda_{{E}}\|\mathbf{E}\|_{*}\\ &+\lambda_{{G}}\|\mathbf{G}\|_{1}+\frac{\beta}{2}\|{ R_{\Omega}(\mathbf{E}-\mathbf{F})}\|^{2}_{F} \\ &+\langle\mathbf{M}_{1}, R_{\Omega}(\mathbf{E}-\mathbf{F})\rangle\\ &+\left\langle\mathbf{M}_{2}, \mathbf{E}- \mathbf{X}^{T} \mathbf{G} \mathbf{Y}-\mathbf{C}\right\rangle\\ &+\frac{\beta}{2}\left\|\mathbf{E}- \mathbf{X}^{T} \mathbf{G} \mathbf{Y}-\mathbf{C}\right\|^{2}_{F} \end{aligned}} $$

where $\mathbf {M}_{1}, \mathbf {M}_{2} \in \mathbb {R}^{m\times n}$ are called Lagrange multipliers and *β*>0 is the penalty parameter. As an iterative algorithm, given **C**^*k*^, **G**^*k*^, $\mathbf {E}^{k}, \mathbf {M}_{1}^{k}$ and $\mathbf {M}_{2}^{k}$ at iteration *k*, we update each group of the variables as follows:

$$\begin{aligned} \mathbf{C}^{k+1}=&\arg\min_{\mathbf{C}}\mathcal{L}\left(\mathbf{E}^{k}, \mathbf{G}^{k}, \mathbf{M}_{2}^{k}, \mathbf{C}\right), \\ \mathbf{G}^{k+1}=&\arg\min_{\mathbf{G}}\mathcal{L}\left(\mathbf{E}^{k}, \mathbf{G}, \mathbf{M}_{2}^{k}, \mathbf{C}^{k+1}\right), \\ \mathbf{E}^{k+1}=&\arg\min_{\mathbf{E}}\mathcal{L}\left(\mathbf{E}, \mathbf{G}^{k+1}, \mathbf{M}_{1}^{k}, \mathbf{M}_{2}^{k}, \mathbf{C}^{k+1}\right). \\ \end{aligned} $$

After solving these subproblems, we update the multipliers **M**_1_ and **M**_2_ as follows;

$$\begin{aligned} \mathbf{M}_{1}^{k+1} = & \mathbf{M}_{1}^{k}+\beta\left(R_{\Omega}\left(\mathbf{E}^{k+1} - \mathbf{F}\right)\right), \\ \mathbf{M}_{2}^{k+1} = & \mathbf{M}_{2}^{k}+\beta\left(\mathbf{E}^{k+1} - \mathbf{X}^{T} \mathbf{G}^{k+1} \mathbf{Y} -\mathbf{C}^{k+1}\right). \end{aligned} $$

Next, we derive the solution to each of the above three subproblems. The four steps are noted as Updating **C**, Updating **G**, and Updating **E**.

Updating **C**: we solve the following problem

$$ \begin{aligned} \min_{\mathbf{C}} &\frac{1}{2}\|\mathbf{C}\|^{2}_{F}+\left\langle\mathbf{M}_{2}^{k}, \mathbf{E}^{k}- \mathbf{X}^{T} \mathbf{G}^{k} \mathbf{Y}-\mathbf{C}\right\rangle\\ &+\frac{\beta}{2}\left\|\mathbf{E}^{k}- \mathbf{X}^{T} \mathbf{G}^{k} \mathbf{Y}-\mathbf{C}\right\|^{2}_{F} \end{aligned} $$

which has a closed form solution as:

$$\mathbf{C}^{k+1} = \frac{\beta}{\beta + 1}\left(\mathbf{E}^{k} - \mathbf{X}^{T} \mathbf{G}^{k} \mathbf{Y} + \mathbf{M}_{2}^{k}/\beta\right) $$

Updating **G**: we need to solve

3 $$ \begin{aligned} \min_{\mathbf{G}} &\lambda_{G}\|\mathbf{G}\|_{1} + \left\langle\mathbf{M}_{2}, \mathbf{E}^{k}- \mathbf{X}^{T} \mathbf{G} \mathbf{Y}- \mathbf{C}^{k}\right\rangle\\ &+\frac{\beta}{2}\left\|\mathbf{E}^{k} - \mathbf{X}^{T} \mathbf{G} \mathbf{Y}- \mathbf{C}^{k}\right\|^{2}_{F}. \end{aligned}  $$

After adding a constant term to Eq. (3), we obtain

$$\min_{\mathbf{G}} \lambda_{G}\|\mathbf{G}\|_{1} +\frac{\beta}{2}\left\|\mathbf{B}^{k}- \mathbf{X}^{T} \mathbf{G} \mathbf{Y}\right\|^{2}_{F} $$

where $\mathbf {B}^{k}=\mathbf {E}^{k}+\mathbf {M}_{2}^{k}/\beta -\mathbf {C}^{k}$. By converting the matrix **G** into a vector **g**=vec(**G**), vec(**X**^*T*^**GY**)=(**Y**^*T*^⊗**X**^*T*^)**g** where ⊗ computes the Kronecker product of two matrices. Further, we let **b**^*k*^=vec(**B**^*k*^). Now, if we denote **A**=(**Y**^*T*^⊗**X**^*T*^), the above problem becomes:

4 $$  \min_{\mathbf{g}} \lambda_{G}\|\mathbf{g}\|_{1} +\frac{\beta}{2}\left\|\mathbf{A} \mathbf{g} - \mathbf{b}^{k}\right\|^{2}_{2}  $$

Equation (4) is a standard least-absolute-shrinkage-and-selection-operator (LASSO) problem, and has to be solved iteratively in practice. It causes a problem to compute or even store **A** because the size of **A** is *nm*×*d*_1_*d*_2_, which is often prohibitively large. Using the stochasticity and linearization techniques in ADMM, we approximate our problem as follows:

5 $$ \begin{aligned} & \frac{1}{2}\left\|\mathbf{A}^{k} \mathbf{g} - \tilde{\mathbf{b}^{k}}\right\|^{2}_{2} \\ \approx & \frac{1}{2}\left\|\mathbf{A}^{k} \mathbf{g}^{k} - \tilde{\mathbf{b}^{k}}\right\|^{2}_{2} + \left\langle{f_{1}^{k}, \mathbf{g} - \mathbf{g}^{k}}\right\rangle + \frac{\tau_{k}}{2}\left\|\mathbf{g}- \mathbf{g}^{k}\right\|^{2}_{2} \end{aligned}  $$

where **A**^*k*^ and $\tilde {\mathbf {b}^{k}}$ contain the data from the corresponding *s* rows of **A** and **b** and the indexes of the *s* rows are randomly drawn from {1,⋯,*nm*}, *τ*_*k*_>0 is a proximal parameter, and

6 $$ \begin{aligned} f_{1}^{k} ={ {\mathbf{A}^{k}}}^{T}\left({\mathbf{A}^{k}} \mathbf{g}^{k} - \tilde{\mathbf{b}^{k}}\right) \end{aligned}  $$

is the stochastic gradient of $\frac {1}{2}\left \|\mathbf {A}^{k} \mathbf {g} - \mathbf {b}^{k}\right \|^{2}_{2}$ at **g**^*k*^. The stochastic approximation can tremendously reduce memory consumption and save computational costs in each iteration. Then Eq. (4) can be approximately re-written as follows by plugging Eq. (5) in Eq. (4):

$$\min_{\mathbf{g}} \lambda_{G}\|\mathbf{g}\|_{1} +\frac{\beta\tau_{k}}{2}\left\|\mathbf{g} - \left[\mathbf{g}^{k}- f_{1}^{k}/\tau_{k}\right]\right\|^{2}_{2} $$

Obviously the closed-form solution is:

$${}\begin{aligned} \mathbf{g}^{k+1}=& \max\left(|\mathbf{g}^{k}- f_{1}^{k}/\tau_{k}|-\frac{\lambda_{G}}{\tau_{k}\beta}, 0\right) \odot sgn\left(\mathbf{g}^{k}- f_{1}^{k}/\tau_{k}\right) \end{aligned} $$

where ⊙ computes the component-wise vector multiplication. Our algorithm calculates each stochastic gradient in parallel by using multiple computation units, i.e., workers, then averaging those gradient values by a central computation unit, i.e., a master. Hence, when solving the subproblem (4) for **G**, we run a parallel stochastic process. Because the term $||\mathbf {A} \mathbf {g} - \mathbf {b}||^{2}_{2}$ is derived from the constraints in the original problem (2), the proposed algorithm actually solves an optimization problem with stochastic constraints.

Updating **E**: we solve the following problem

$$\begin{aligned} & \min_{\mathbf{E}}\lambda_{E}\|\mathbf{E}\|_{*}+\left\langle\mathbf{M}^{k}_{1}, R_{\Omega}(\mathbf{E}-\mathbf{F})\right\rangle + \frac{\beta}{2}\|R_{\Omega}(\mathbf{E}-\mathbf{F})\|^{2}_{F} \\ & +\left\langle\mathbf{M}^{k}_{2}, \mathbf{E}- \mathbf{X}^{T} \mathbf{G}^{k+1} \mathbf{Y} - \mathbf{C}^{k}\right\rangle\\ &+\frac{\beta}{2}\left\|\mathbf{E}- \mathbf{X}^{T} \mathbf{G}^{k+1} \mathbf{Y} - \mathbf{C}^{k}\right\|^{2}_{F}, \end{aligned} $$

and we can re-organize this subproblem into a simpler form as:

$$\min_{\mathbf{E}}\lambda_{E}\|\mathbf{E}\|_{*}+\frac{\beta}{2}\|R_{\Omega}\left(\mathbf{E}-\mathbf{B}_{2}^{k}\right)\|^{2}_{F} +\frac{\beta}{2}\|\mathbf{E}- \mathbf{B}_{3}^{k}\|^{2}_{F} $$

where $\mathbf {B}_{2}^{k}=R_{\Omega }\left (\mathbf {F}-\mathbf {M}_{1}^{k}/\beta \right)$ and $\mathbf {B}_{3}^{k}=\mathbf {X}^{T} \mathbf {G}^{k+1} \mathbf {Y}+\mathbf {C}^{k} - \mathbf {M}_{2}^{k}/\beta $. By the same linearization technique used in Updating **G**, the problem can be approximated by:

$$\begin{aligned} \min_{\mathbf{E}}\lambda_{E}\|\mathbf{E}\|_{*} + \frac{\beta \tau^{\prime}_{k}}{2} \left\|\mathbf{E}-\left(\mathbf{E}^{k}-f_{2}^{k}/\tau'_{k}\right)\right\|^{2}_{F} \\ + \frac{\beta \tau^{\prime}_{k}}{2} \left\|\mathbf{E}-\left(\mathbf{E}^{k}- f_{3}^{k}/\tau^{\prime}_{k}\right)\right\|^{2}_{F} \end{aligned} $$

where $f_{2}^{k}$ and $f_{3}^{k}$ are the gradients of $\frac {1}{2}\left \|R_{\Omega }\left (\mathbf {E}-\mathbf {B}_{2}^{k}\right)\right \|^{2}_{F}$ and $\frac {1}{2}\left \|\mathbf {E}- \mathbf {B}_{3}^{k}\right \|^{2}_{F}$ at **E**^*k*^, respectively, which can be computed as follows:

7 $$ \begin{aligned}  f_{2}^{k} & = R_{\Omega}\left(\mathbf{E}^{k}-\mathbf{B}_{2}^{k}\right)= R_{\Omega}\left(\mathbf{E}^{k} - \mathbf{F} + \mathbf{M}_{1}^{k}/\beta\right),\\ f_{3}^{k} & = \mathbf{E}^{k}-\mathbf{B}_{3}^{k}= \mathbf{E}^{k} - \mathbf{X}^{T} \mathbf{G}^{k+1} \mathbf{Y} - \mathbf{C}^{k} + \mathbf{M}_{2}^{k}/\beta. \end{aligned}  $$

Therefore, the closed-form solution can be obtained as

$$\begin{aligned} \mathbf{E}^{k+1}& = SVT\left(\mathbf{E}^{k} - \left(f_{2}^{k}+f_{3}^{k}\right)/\left(2\tau'_{k}\right), \lambda_{E}/2\left(\beta\tau'_{k}\right)\right) \end{aligned} $$

Here the operator *SVT*(**E**,*t*) is defined in [12] for thresholding the singular values of a matrix **E** by *t* (i.e., only keeping the singular values of **E** greater than or equal to *t* and setting others to 0).

Algorithm 1 summarizes the StoLADMM steps for optimizing the variables (**C**,**E**,**G**,**M**_1_,**M**_2_).


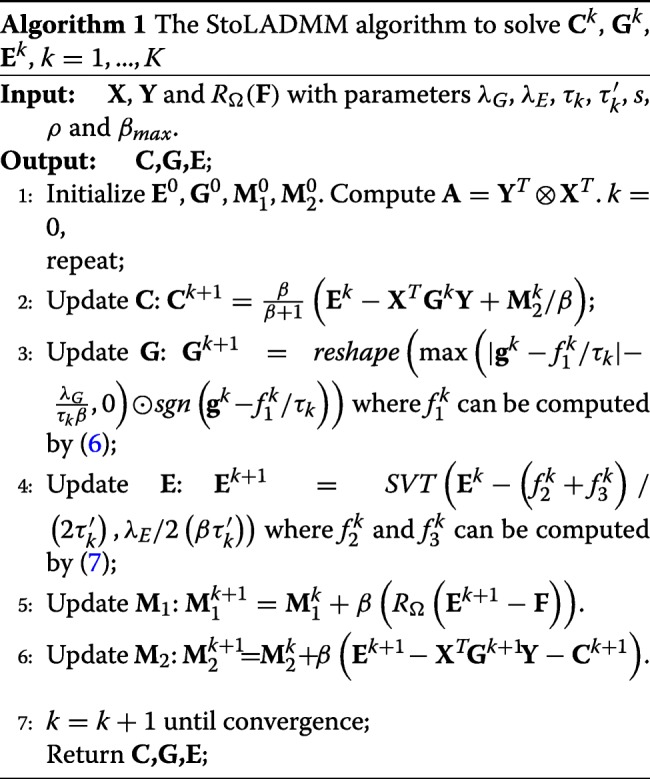


It can be proven that the proposed algorithm, which belongs to the family of stochastic ADMM methods, has an $O(1/\sqrt {k})$ convergence rate [32], while achieving both storage and computational efficiency. When running Algorithm 1, we set the sampling block size *s* to be $\max {(1, \sqrt {\text {length}(\mathbf {g})/100})}$, and *τ*_*k*_<∥**A**∥, $\tau ^{\prime }_{k}<\|R_{\Omega }(\mathbf {F})\|$ and *β* = 0.01 as the preferable values listed in [20, 34]. In the initialization step, $\mathbf {M}^{0}_{1}$ and $\mathbf {M}^{0}_{2}$ are randomly drawn from the standard Gaussian distribution; we initialize **E**_0_ and **G**_0_ by the iterative soft-thresholding algorithm [35] and *SVT* operator respectively. In addition to the convergence property and computational efficiency, our algorithm improves its usability by application of the linearization technique because two of the subproblems are non-smooth with the *ℓ*_1_-norm or the nuclear norm, and are difficult to solve without the linearization and thresholding.

Despite the recent intensive studies on stochastic optimization algorithms such as the stochastic gradient descent [36, 37] and stochastic ADMM [32, 33], much less work has addressed optimization problems with a large number of constraints. The most related work is the method in [38, 39] where a primal-dual stochastic algorithm was proposed for constrained optimization and attained an optimal convergence rate of $O(1/\sqrt {k})$ for Lipschitz continuous objectives; an online optimization algorithm was used in [39] where the objective function consisted of a Lyapunov drift term and an online penalty term. However, none of these methods investigated ADMM methods for stochastic constraints.

## Results

To test the effectiveness and scalability of the proposed algorithm, we first experimented with completing synthetic matrices of various sizes, and compared the method against other state-of-the-art matrix completion approaches. Then, we used the method to analyze our Opioid-Cocaine SUD Dataset. This dataset was created by aligning the 11 diagnostic criteria for CUD and the 11 criteria for OUD for all 3,441 patients to form **F**. The competing methods that also used side information included: LADMM [40], MAXIDE [17], IMC [16] and DirtyIMC [18]. The performance of all methods was measured by the relative mean squared error (RMSE) calculated on the missing entries: $\|R_{\not \Omega }\left (\mathbf {X}^{T} \mathbf {G} \mathbf {Y} - \mathbf {F}\right)\|^{2}_{2} / \|R_{\not \Omega }(\mathbf {F})\|^{2}_{2}$.

The rank of **G** was a hyperparameter required by IMC and DirtyIMC and the regularization hyperparameters *λ*’s were used by all methods. We first left out a portion (*q%*) of data in **F** for the final testing. We ran cross-validation within the remaining data to determine *λ*’s: we randomly drew 30% of the given entries of **F** as a validation set. Then each model was constructed using the remaining entries with different *λ* choices from 10^−3^,10^−2^,...,10^4^. For IMC and DirtyIMC, the best rank of **G** was chosen from 1 to 15 within each 30–70% data split. Experiments with each hyperparameter setting were repeated three times and the average RMSE was calculated. The hyperparameter values that gave the best average validation RMSE were chosen for each individual method.

In our experiments, we repeated the entire procedure 5 times and reported the average RMSE on the missing *q%* entries (i.e., the test RMSE). The procedure for removing the *q%* of entries in **F** is described separately in the simulations and in our case study. All tests were conducted using Matlab and experiments were performed on an Intel Core i7 3.6GHz computer with 16GB RAM.

### Simulations

We created synthetic matrices of 200×200 and 1000×1000. Note that the 1000×1000 matrix corresponded to a large dataset of 10^6^ entries. To mimic real-world complexity, we synthesized data for each feature in both **X** and **Y** according to a distribution that was randomly selected from Gaussian, Poisson and Gamma distributions. To generate **G**, the location of the non-zero entries of **G** were randomly selected and their values were drawn from a Gaussian distribution $\mathcal {N}(0, 100)$ independently and identically, which we repeated several times to choose the matrices that were full- or high-rank. We then generated **F** by computing **F**=**X**^*T*^**GY**+**N** where **N** represented noise and each component in **N** was drawn from $\mathcal {N}(0, 1)$. We used $\mathcal {N}(0, 1)$ to create noise so the larger signals in **G** drawn from $\mathcal {N}(0, 100)$ had enough chance to be recognized. Then *q* percent of the entries in **F** were randomly drawn and set to be missing. For each simulated **F** matrix, we ran all methods with multiple choices of missing data amount, and we used *q* ∈[ 10*%* − 90*%*] and a step size of 10%.

**Fig. 2:**
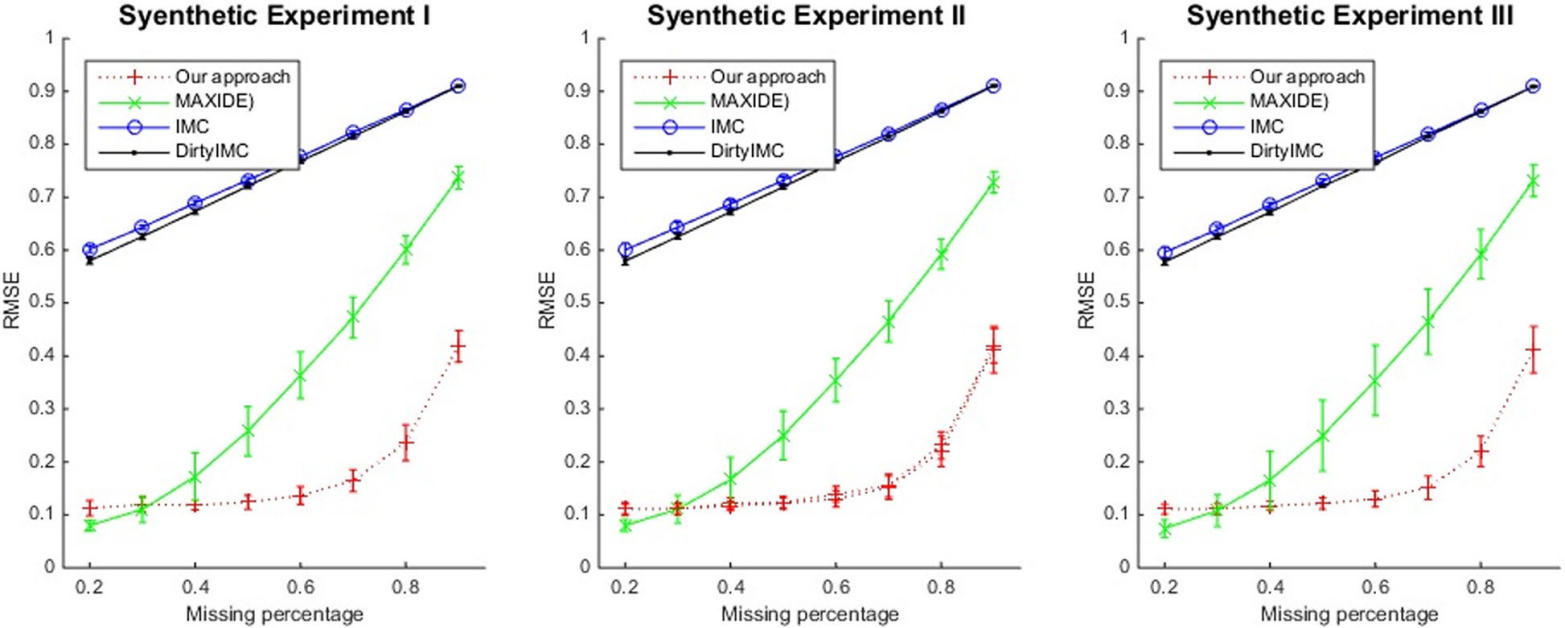
The Comparison of the average RMSE values and standard deviations as bars in Synthetic Experiments I, II, and III

We compared the different methods in the three synthetic experiments I, II and III. In the first setting, the dimension of **X** and **Y** was set to 15×200 and 20×200 and all features in these two matrices were randomly generated (in the same procedure as the generation of **G**) to make them full row rank. In the other two settings, **X** and **Y** were not full row rank. The dimension of **X** and **Y** was set to 16×200 and 21×200 in the setting II, and 20×1000 and 25×1000 in the setting III, respectively. For these two settings, the first 15 features in **X** and 20 features in **Y** were randomly created, but the remaining features were generated by computing linear combinations of the random created features. We generated 10 synthetic datasets for each setting using the same procedure as described above and reported the mean and standard deviation of test RMSE values, which are shown in Fig. 2.

**Fig. 3:**
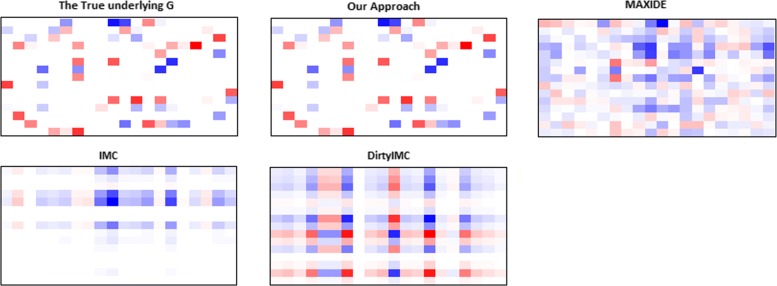
The heatmap of the true G and recovered G matrices in Synthetic Experiment I

Based on Fig. 2, our approach achieved greater accuracy than the other methods in all the different settings. As the missing percentage *q* grew, the RMSE of our method increased to a lesser degree than that of other methods. We reviewed the ranks of the recovered **G** and **E** in the first setting. For each method, the **G** and **E** matrices that achieved the best performance were examined. The ranks of **G** and **E** from our method, MAXIDE, IMC, DirtyIMC were 15, 8, 1, 1 and 15, 7, 1, 1, respectively. Thus, our method appeared to recover the interactive matrix **G** more accurately than the other methods, probably because the fact that other methods used an unnecessarily strong prior of low rank **G**. We calculated and showed the recovered model matrices **G** for all of the methods at the missing percentage of *q*= 50% and compared them with the true **G** in Fig. 3. As can be seen there, our method was the only one that could recover the true **G**.

To empirically validate the scalability of our method, Table 2 lists the run time in seconds and accuracies of all the competing methods including the non-stochastic LADMM algorithm on synthetic matrices with the size of 1000×1000 in another Synthetic Experiment IV. The result showed that accelerating the method did not sacrifice the final recovery accuracy noticeably. Although the proposed algorithm used only 5% of the time taken by the non-stochastic LADMM, meaning 20 times faster than the standard LADMM algorithm, the imputation accuracy as measured by RMSE was better than the other methods. These observations demonstrate that our stochastic method can be a better alternative to handle big datasets.

**Table 2:** The Comparison of RMSE values and computation time of different methods in Synthetic Experiment IV

*q*		StoLADMM	LADMM	DirtyIMC	IMC	MAXIDE
10%	RMSE	0.061	0.062	0.419	0.402	-
	time(s)	1.773	20.727	0.827	4.750	-
20%	RMSE	0.095	0.098	0.453	0.468	-
	time(s)	1.475	26.781	0.757	4.297	-
30%	RMSE	0.085	0.076	0.499	0.402	-
	time(s)	1.447	18.807	0.406	4.750	-
40%	RMSE	0.089	0.069	0.593	0.620	-
	time(s)	1.452	18.976	0.420	4.796	-
50%	RMSE	0.081	0.076	0.716	0.700	-
	time(s)	1.382	22.057	0.248	3.156	-

Computation time is measured by seconds, and ‘-’ represents running failure, i.e., the method fails due to the out-of-memory issue

### Inference of CUD and OUD diagnostic criteria

We used the proposed approach to analyze the data of 3441 SUD subjects for whom both CUD and OUD diagnostic criteria were recorded, which means that we had a fully observed matrix **F**. To mimic the real-life situation where the use of a substance might not be reported, thus missing all criteria for that substance, we randomly selected *q* percent of SUD patients, for whom we removed randomly either CUD or OUD diagnostic criteria. We evaluated the performance with 5 different *q* values: 20%, 40%, 60%, 80%, and 100%. Note that when *q*= 100%, every patient had either CUD or OUD diagnostic criteria removed but not both. There were 383 genetic variants selected in our GWAS, which were used as side information in **X**. We computed the correlations between each pair of the 22 criteria using all patients and used the correlation matrix as **Y**.

In addition to the four competing methods used in the simulations, we also compared our method to a naive method (NM) in which the missing criteria of a disorder were filled by copying over the patient’s diagnostic symptoms for the other substance. The proposed algorithm was evaluated using the same training and tuning procedure as used in the simulations. The imputation accuracy and computation time of all methods are shown in Table 3. Because there was no imputation in the NM method, run time was not given in the table. The best performance was again obtained by our approach in terms of both accuracy and time efficiency in comparison with other imputation methods.

**Table 3:** The comparison of imputation results by different methods on the Opioid-Cocaine SUD dataset

*q*		StoLADMM	LADMM	DirtyIMC	IMC	MAXIDE	NM
20%	RMSE	0.236	0.231	0.297	0.230	0.235	0.567
	time(s)	30.938	664.515	45.366	21.053	4732.718	-
40%	RMSE	0.226	0.234	0.298	0.235	0.236	0.582
	time(s)	29.953	982.212	21.063	20.803	3772.202	-
60%	RMSE	0.228	0.236	0.301	0.237	0.235	0.581
	time(s)	28.719	815.841	20.269	36.737	4718.916	-
80%	RMSE	0.236	0.237	0.303	0.239	0.241	0.585
	time(s)	30.547	877.886	23.906	32.872	4011.692	-
100%	RMSE	0.223	0.239	0.303	0.246	0.242	0.574
	time(s)	30.172	489.770	22.922	24.653	3695.292	-

**Fig. 4:**
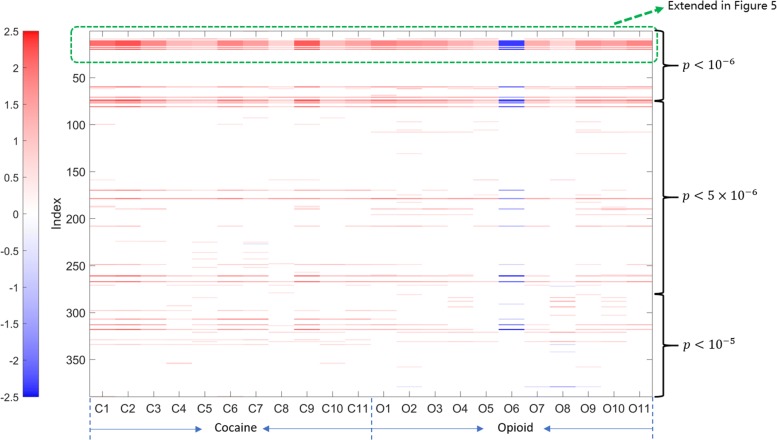
The recovered **G** by our method for the Cocaine-Opioid SUD dataset. Columns C1-C11 represent 11 CUD diagnostic criteria, columns O1-O11 represent 11 OUD diagnostic criteria. C1/O1: Larger or longer Cocaine/Opioid use than intended; C2/O2: Failed efforts to stop on Cocaine/Opioid; C3/O3: Much time spent in Cocaine/Opioid related activities; C4/O4: Strong desire to use Cocaine/Opioid; C5/O5: Cocaine/Opioid effect interfered with life; C6/O6: Cocaine/Opioid use despite of its interference; C7/O7: Major activities reduced by Cocaine/Opioid use; C8/O8: Physical hazard caused by Cocaine/Opioid use; C9/O9: Cocaine/Opioid use knowing it threatening health; C10/O10: Cocaine/Opioid tolerance; C11/O11: Cocaine/Opioid withdrawal syndrome

**Fig. 5:**
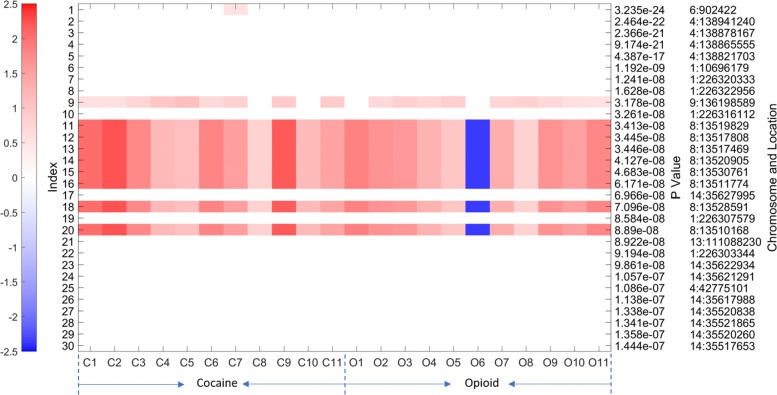
The top 30 rows of the recovered **G** by our method for the Cocaine-Opioid SUD dataset. Columns correspond to the diagnostic criteria for CUD and OUD whereas rows correspond to the candidate genetic variants. The right-hand side gives the locations of these genetic variants and their *p*-values obtained in the GWAS

Figure 4 shows the parameter matrix **G** (of size 383×22) obtained by our algorithm. Note that the genetic variants were ordered in ascending fashion with respect to their association *p*-values reported in the GWAS, so the most significant variants identified in the GWAS are at the top of the figure. A more saturated color reflects a stronger interaction between a specific genetic variant and a diagnostic criterion. Red denotes positive interactions and blue denotes negative interactions. We further expanded first 30 rows of Fig. 4 into Fig. 5. It can be observed from Figs. 4 and 5 that the first 30 most significant variants from the GWAS had the largest magnitude interactions with the criteria. Another observation on Fig. 4 is that genetic variants with lower (stronger) association *p*-values are more likely to show stronger interactions with the phenotypes.

## Discussion

**Fig. 6:**
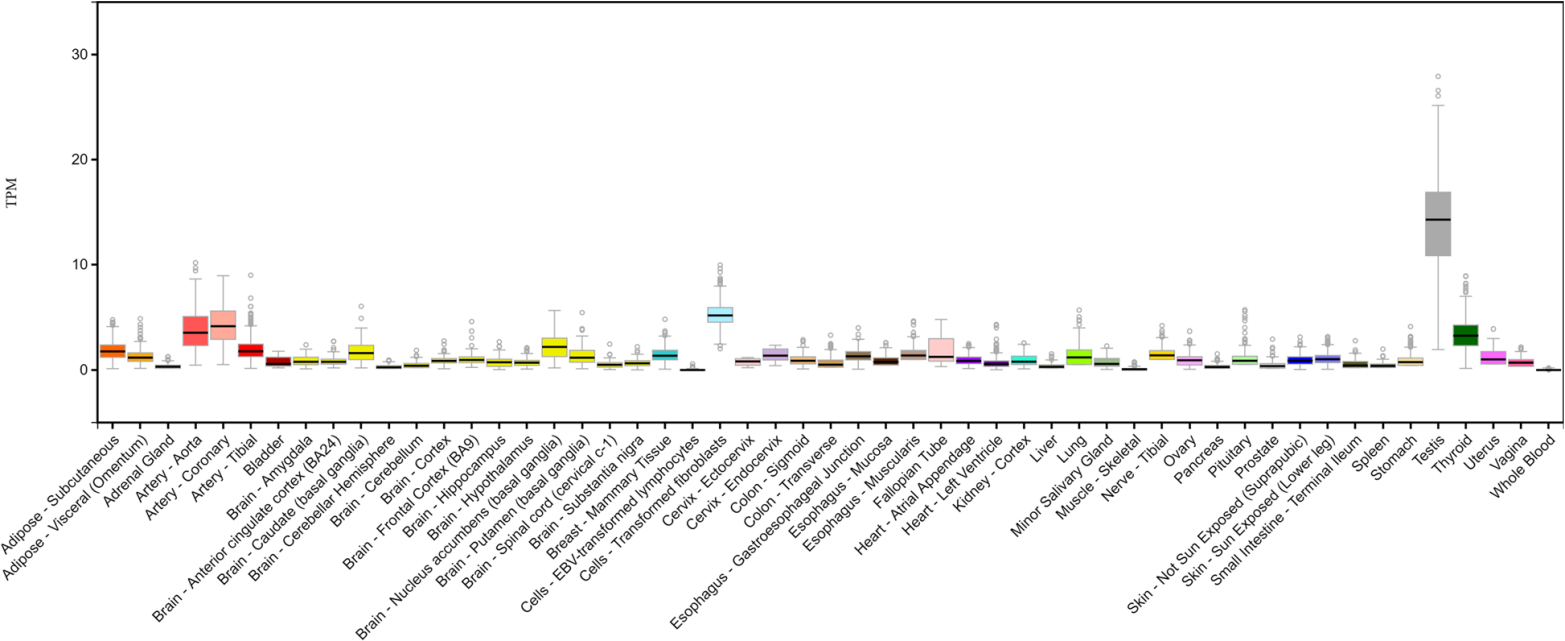
Gene expression distribution (RPKM, Reads per Kilobase Million) of C8orf48 across human tissues

In this section, we discuss other benefits besides the accuracy and efficiency of the proposed approach. In Fig. 5, 9 of the variants and their interactions with diagnostic criteria received high weights when imputing the unreported criteria. It is also interesting to observe that the interactions between all these variants and the opioid diagnostic criterion “opioid use despite its interference” were negatively proportional to the imputed values of missing criteria for CUD, which may need further investigation in a future study. The SNP rs1481605 at base pair (bp) 13,519,829 on chromosome 8 received the highest weights for its interactions with all 22 phenotypes in the model. Moreover, this SNP was associated with both OUD and CUD at genome-wide significant level (*p*<5×10^−8^) in the GWAS. This SNP is located at the downstream (94,032 bp away) of gene *C8orf48*, which, according to data from GTEx (available at https://www.gtexportal.org/home/), expresses in many brain tissues, and its expression in nucleus accumbens is the highest, as illustrated in Fig. 6 copied from the GTEx website.

## Conclusion

In conclusion, we have proposed a new approach based on a matrix completion technique that uses genotype data to infer diagnostic criteria of a disorder, specifically, diagnostic criteria of substance use disorders. Our approach can integrate side information at different scales extending from the DNA scale to the behavioral scale (derived from other comorbid disorders). By imposing a sparse prior on the model parameter matrix **G**, the method can help to identify important interactions that link specific genotypes to diagnostic criteria. An efficient stochastic LADMM algorithm has been developed to solve the related optimization problem 5% of the time required by the non-stochastic algorithm. Experimental evaluation of the proposed approach shows that it outperforms the state-of-the-art for phenotype inference by improving both accuracy and computational efficiency. These results also demonstrate that effectively integrating genotype data with other relevant sources of information is a better alternative for imputing missing phenotypes than using a single source. As an additional benefit, the proposed method constructs a bi-linear predictive model that can be used to predict symptoms of new subjects more effectively than classical low rank matrix completion methods, which do not produce a model.
